# Editorial: Host/Parasite Molecular and Cellular Interactions in the Establishment and Maintenance of Protozoan Infections

**DOI:** 10.3389/fcimb.2022.930073

**Published:** 2022-05-24

**Authors:** Martin M. Edreira, Maria E. Francia, Natalia de Miguel

**Affiliations:** ^1^Laboratorio de Biología Molecular de Trypanosomas, IQUIBICEN, CONICET-Universidad de Buenos Aires, Ciudad de Buenos Aires, Argentina; ^2^Departamento de Química Biológica, Facultad de Ciencias Exactas y Naturales, Universidad de Buenos Aires, Ciudad de Buenos Aires, Argentina; ^3^Department of Pharmacology and Chemical Biology, School of Medicine, University of Pittsburgh, Pittsburgh, PA, United States; ^4^Laboratory of Apicomplexan Biology, Institut Pasteur de Montevideo, Montevideo, Uruguay; ^5^Departamento de Parasitología y Micología, Facultad de Medicina, Universidad de la República, Montevideo, Uruguay; ^6^Laboratorio de Parásitos Anaerobios, Instituto Tecnológico de Chascomús (CONICET-UNSAM), Chascomús, Argentina; ^7^Escuela de Bio y Nanotecnologías, Universidad Nacional de San Martin (UNSAM), Buenos Aires, Argentina

**Keywords:** Apicomplexa, Kinetoplastid, Anaerobic parasites, host-pathogen interaction, immune evasion, host cell subversion

## Introduction

Protozoan parasites cause several human and veterinary diseases such as leishmaniasis, malaria, trypanosomiasis, toxoplasmosis, trichomoniasis and amebiasis, resulting in considerable morbidity and mortality worldwide, particularly affecting low-income countries. To establish successful infections in their hosts, protozoan parasites have developed a wide range of strategies. However, despite being of critical importance, the cellular and molecular mechanisms underlying host/parasite interactions are still not well understood.

Herein we briefly introduce a collection of 16 original research articles and literature reviews written by over 100 authors, reflecting the state of the art in host-pathogen interactions, highlighting different cellular and molecular mechanisms employed by protozoan parasites to establish a successful infection and thrive within their hosts

## Multi-Species Host- Parasites Interaction

Parasite encoded Nucleotidases or NTPDases catalyze the hydrolysis of ATP to ADP. These cell-surface hydrolases have been shown to play multiple roles in parasite’s infectivity and virulence by impacting purine salvage pathways and parasite adhesion to the host cell. The roles of NTPDases in parasite biology and their potential as drug targets in multiple species, including *Leishmania* spp., *Trypanosoma* spp.*, Trichomonas vaginalis, Toxoplasma gondii* and *Schistosoma*, are reviewed by Paes-Vieira et al. This review highlights the promise of devoting efforts to improving the selectivity and specificity of compounds assayed against E-NTPDases from different parasites, as promising roads to elimination.

## Host-Apicomplexan Parasites Interactions

Apicomplexans, including parasites of the *Plasmodium* genus are obligate intracellular parasites. Different life forms of the *Plasmodium* parasites bear cell type specificity, with sporozoites invading liver cells and merozoites red blood cells, but it’s unknown how the parasite distinguishes between cell types. Based on transcriptome analysis of hepatoma cells, which are refractory to parasite invasion, Amanzougaghene et al. postulate that the transmembrane protein Aquaporin-9 (AQP9) plays a significant role in facilitating *Plasmodium*’s invasion. AQP9 overexpression in hepatocytes increases their permissiveness to *P. falciparum*, while its downregulation increases their refractoriness. Interestingly, the dependence on AQP9 for invasion was shown to be species-specific.

While specific parasite proteins play essential roles in the invasion and survival within the host, metabolic pathways present in the host, which have co-evolved with the parasites, provide unexplored opportunities for intervention. It is well established that the outcome of clinical malaria is interdependent on erythrocyte metabolism. Chronic metabolic disorders affecting erythrocytes are positively selected in malaria endemic areas. However, the role of acute inhibition of metabolic pathways as plausible treatment strategies have not been extensively evaluated. Erythrocytes particularly rely on the enolase isozyme ENO2 for glycolytic function. Jezewskiet al., explore the effects of specific ENO2 inhibition by non-prodrugged phosphonates on parasite viability. They show that the significant antimalarial potency of each of three assayed compounds was highly correlated to their individual capacity to disrupt erythrocyte redox balance *in vitro*, and their degree of inhibitor-induced anemia *in vivo* in mice. Interestingly multi-resistant strains to antimalarials are sensitive to ENO2 inhibition).

Tumor necrosis factor (TNF) is a major inflammatory cytokine known to play pivotal roles in the responses mounted to parasitic infections, including those caused by apicomplexan and kinetoplastids parasites. Batista Ferreira França et al. evaluate the role of TNF in the cellular and humoral immune responses to the bovine abortigenic parasite *Neospora caninum*. Using mice lacking the TNF receptor, TNFR1, the authors demonstrate that lack of TNF induced signaling causes increased inflammation, increased parasite burden in the brain, whilst it reduces nitric oxide (NO) levels and IgG1 production. Overall, this study highlights the importance of TNF - TNF receptor 1 signaling during *N. caninum* infection.

## Host-Kinetoplastid Parasites Interactions

The molecular underpinnings of host immune response subversion are highlighted in many studies included in this topic. Freitas-Mesquita and Meyer-Fernandes review the multiple roles of stage-specific class I nucleases of the *Leishmania* genus in subverting the host-cell defense. In addition, Bichiou et al. explore how *Leishmania* infection modulates macrophage response. Downstream targets of nuclear erythroid related factor 2 (Nrf2) are enzymes related to tolerance of the oxidative stress produced by the oxidative burst required to fight pathogens. The authors analyze the expression levels of NRF2 and its target genes in bone marrow derived macrophages isolated from *Leishmania*-resistant and *Leishmania*-susceptible mice (BMdMs), infected with *L. major* promastigotes. The study uncovers that *Leishmania* infection strongly induces the expression of NRF2 but does not affect the expression of heme oxygenase 1 (HO-1), a factor whose levels are known to affect parasite survival, in mice of different genetic backgrounds. Despite the induction of NRF2, Bichiou et al., report that the transcription of glutathione reductase (Gsr) and cysteine/glutamate exchange transporter (Slc7a11), involved in glutathione accumulation, are actively repressed in infected (susceptible) Balb/c BMdMs. In line with this observation, the study shows that silencing of NRF2 increases the survival and multiplication of the parasite, weakening the cell’s ability to control the infection (Bichiou et al., 2021). In the case of *T. cruzi*, Somoza et al., demonstrate that trypomastigotes directly stimulated B cells that can module the development of a proinflammatory response by impairing their proliferation and inducing an apoptotic process in T CD4+ cells.

In addition to overcoming the immune system, and in order to establish a productive infection, different signaling pathways are activated by *T. cruzi* prior host cell invasion. Manchola Varón, et al., describe the involvement of calcium signaling in parasite interaction with the extracellular matrix at an early stage, and the organelles involved in calcium homeostasis during this interaction.

Extracellular vesicles, secreted surface proteins and enzymes involved in metabolism of *T. cruzi* have been shown to influence virulence and host cell invasion. Four original articles in this series tackle de roles of several *T. cruzi* encoded proteins, assaying their role in parasite survival. Loch et a.l., examine the differential release of gp82 and gp90 as well as their involvement in the shedding process and regulation of host cell invasion. Texeira et al., demonstrate that P21, a secreted protein with immunomodulatory properties, plays a role in facilitating invasion, but appears to negatively regulate amastigote proliferation in mammalian cells. Dick et al., have identified TcIT, a putative 39-kDa Fe transporter, and present evidence regarding its participation in Fe metabolism, proliferation/differentiation, virulence, and maintenance of *T. cruzi* infection. Finally, Silva Oliveira et al. reveal that changes in *T. cruzi* surface molecules are induced by the intracellular environment, which could confer adaptability to the parasite allowing it to infect specific tissues.

It is interesting to note that *T. cruzi* has shown to be a remarkably heterogeneous taxon, presenting extensive inter- and intrastrain genetic diversity and differential infectivity. Rodrigues Cortez et al., designed intraspecific array-based on comparative genomic hybridization to identify chromosomal regions harboring copy-number variations between two strains. The authors discuss the genome plasticity which responds to environmental stress by varying gene copy number and generating segmental aneuploidy.

## Host-Anaerobic Parasites Interactions

Protozoan parasites that reside extracellularly and in oxygen-deprived environments (anaerobic) within their hosts include enteric human pathogens such as *Giardia intestinalis*, *Entamoeba histolytica*, and the human and bovine urogenital tract parasites *Trichomonas vaginalis* and *Trichomonas foetus*, respectively. In this Research Topic, Chadha and Chadee present an update of the current situation regarding *Entamoeba histolytica* and other protozoan parasites that induce outside-in and inside-out signaling to modulate NF-κB in disease pathogenesis and survival in the host. Additionally, Galindo et al., describe the role of *E. histolytica* ESCRT-I complex in vesicular trafficking and phagocytosis, as well as, the relationship between molecules involved in phagocytosis. The authors also demonstrate that phagocytosis is an important step in the mechanism of aggression by *E. histolytica* trophozoites during tissue invasion. Finally, Garzon et al., introduce the use of immunoinformatic approaches to identify and characterize potential T-cell and B-cell epitopes of *Giardia* immunogenic proteins. This bioinformatics analysis provides a deeper understanding of the *Giardia* immunogens that might bind to critical molecules of the host immune system. This approach could help develop novel strategies of peptide-based vaccines against giardiasis.

## Closing Remarks

We hope that the research showcased in this Research Topic highlights the importance of conceiving parasitic infections comprehensively within their natural context. The understanding of the mechanisms of pathogenesis is essential to unravel the complex co-evolved host-parasite biology underlying the deadly diseases highlighted herein, and could lead to effective rational strategies for the design of new and much needed therapies. Host-parasite interactions should remain a research priority in veterinary medicine and public health.

## Author Contributions

ME, MF, and ND drafted the manuscript. All authors contributed to manuscript revision, read, and approved the submitted version.

## Conflict of Interest

The authors declare that the research was conducted in the absence of any commercial or financial relationships that could be construed as a potential conflict of interest.

## Publisher’s Note

All claims expressed in this article are solely those of the authors and do not necessarily represent those of their affiliated organizations, or those of the publisher, the editors and the reviewers. Any product that may be evaluated in this article, or claim that may be made by its manufacturer, is not guaranteed or endorsed by the publisher.

